# Supplementary material for: Associations between the microbiome and immune responses to an adenovirus-based HIV-1 candidate vaccine are distinct between African and US cohorts
Source: mSystems. 2026 Jan 15;11(2):e01435-25. doi: 10.1128/msystems.01435-25 (PMC12911364; doi:10.1128/msystems.01435-25)
Supplement: Supplemental Figures Part 1 — Figures S1 to S4. [file msystems.01435-25-s0001.pdf]

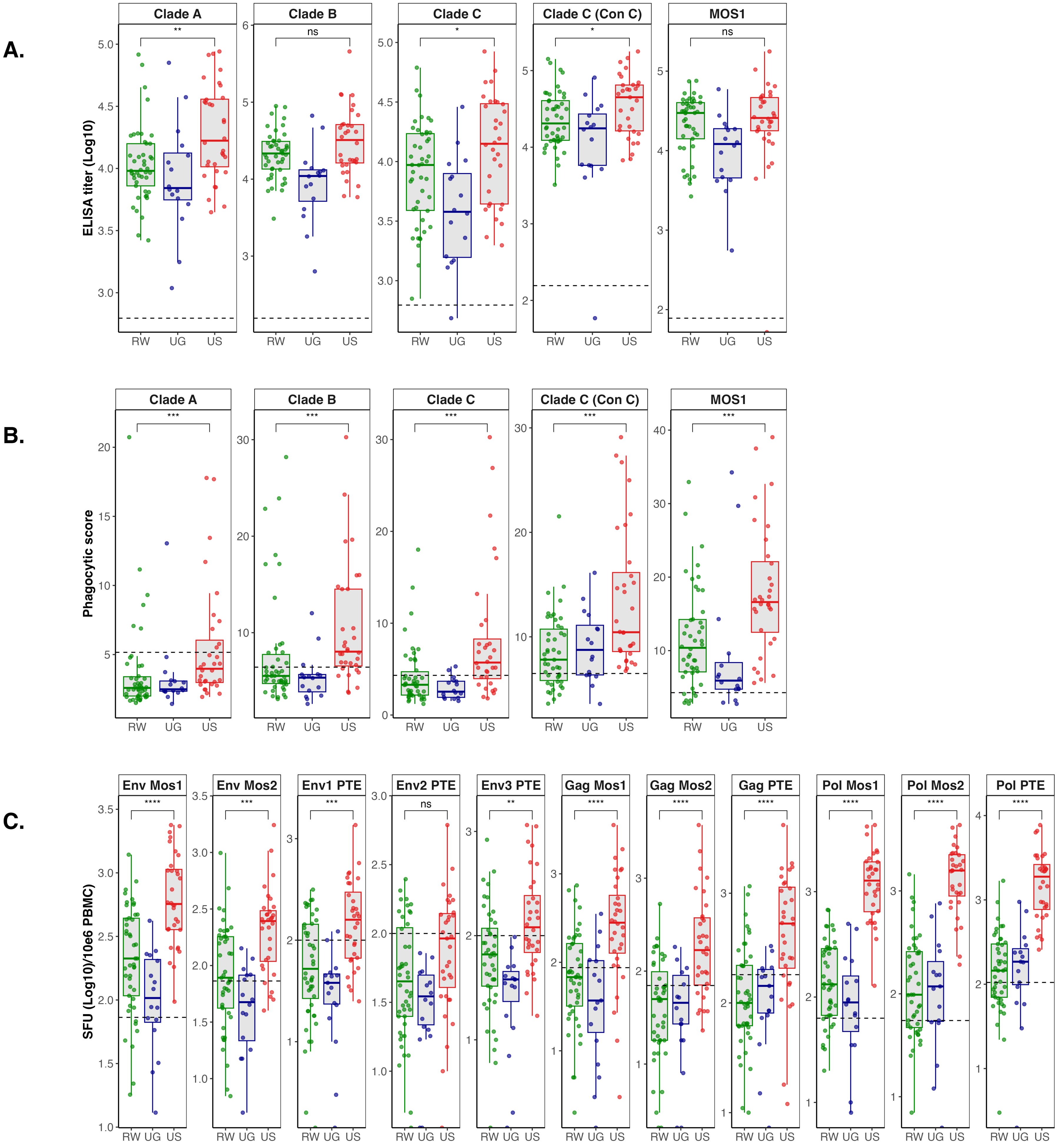

A.
Clade A
Clade B
Clade C
Clade C (Con C)
MOS1
ELISA titer (Log10)
**
ns
*
*
ns
RW
UG
US
B.
Clade A
Clade B
Clade C
Clade C (Con C)
MOS1
Phagocytic score
***
RW
UG
US
C.
Env Mos1
Env Mos2
Env1 PTE
Env2 PTE
Env3 PTE
Gag Mos1
Gag Mos2
Gag PTE
Pol Mos1
Pol Mos2
Pol PTE
SFU (Log10)/10e6 PBMC)
****
***
ns
**
RW UG US

Supplemental Figure 1. Vaccine-elicited immune responses are stronger in participants from the US compared to participants from East Africa. Humoral immune responses to clade-specific antigens were measured in samples collected in the US, Rwanda (RW) and Uganda (UG) by **(A)** ELISA and **(B)** ADCP assays, and cellular immune responses were measured by **(C)** ELISpot assay. Data for all vaccinated participants, excluding those who received placebo, from **Figure 1A** are included. Significance was determined using the Dunn's Test and adjusted for multiple comparisons using the Benjamini-Hochberg method. ****P < 0.0001; ***P < 0.001; **P < 0.01; *P < 0.05; ns = not significant.

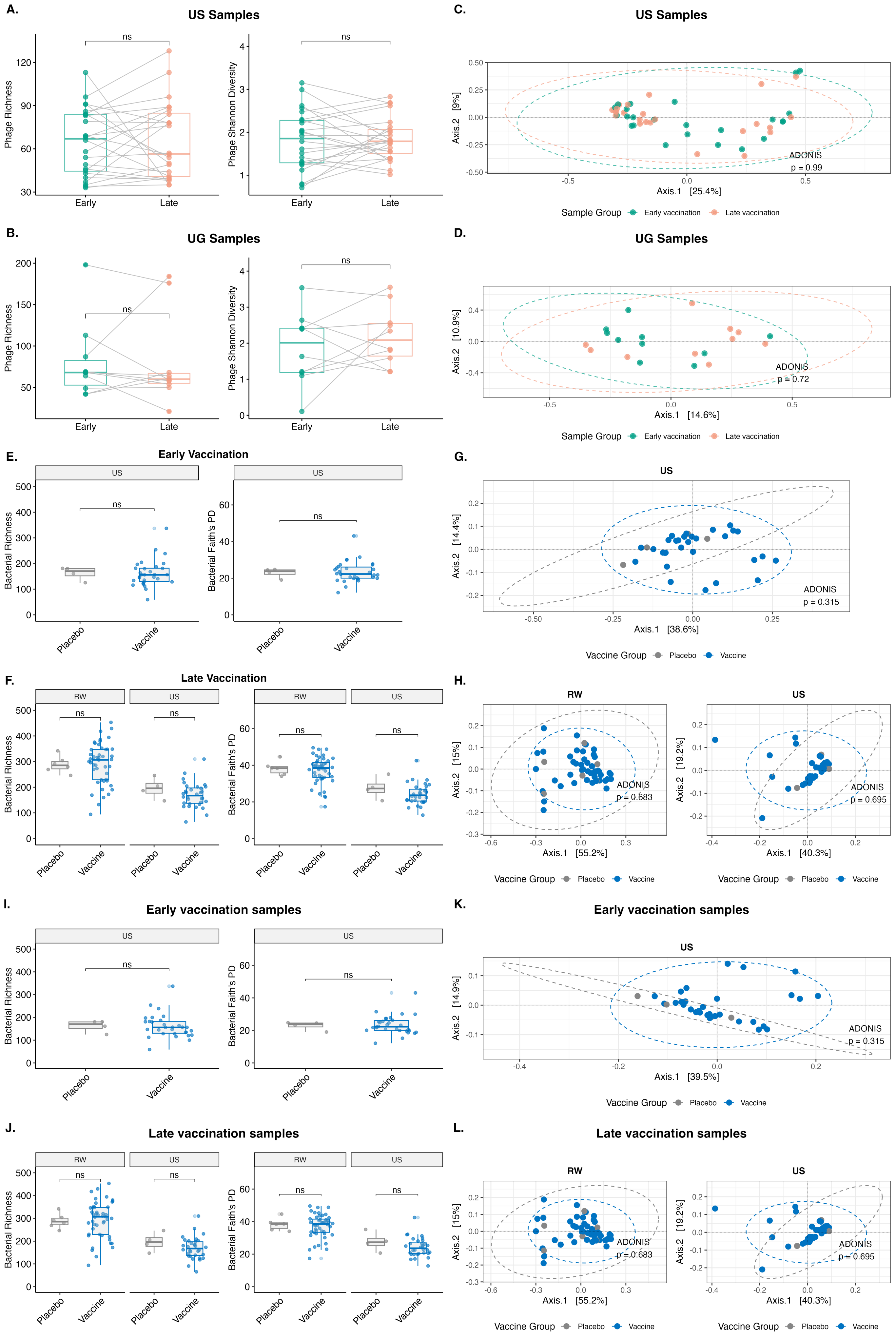
A.
US Samples
Phage Richness
Phage Shannon Diversity
Early
Late
ns
C.
US Samples
ADONIS
p = 0.99
Axis.1 [25.4%]
Axis.2 [9%]
Sample Group
Early vaccination
Late vaccination
B.
UG Samples
Phage Richness
Phage Shannon Diversity
Early
Late
ns
D.
UG Samples
ADONIS
p = 0.72
Axis.1 [14.6%]
Axis.2 [10.9%]
Sample Group
Early vaccination
Late vaccination
E.
Early Vaccination
US
Bacterial Richness
Bacterial Faith's PD
Placebo
Vaccine
ns
G.
US
ADONIS
p = 0.315
Axis.1 [38.6%]
Axis.2 [14.4%]
Vaccine Group
Placebo
Vaccine
F.
Late Vaccination
RW
US
Bacterial Richness
Bacterial Faith's PD
Placebo
Vaccine
ns
H.
RW
US
ADONIS
p = 0.683
p = 0.695
Axis.1 [55.2%]
Axis.2 [15%]
Axis.1 [40.3%]
Axis.2 [19.2%]
Vaccine Group
Placebo
Vaccine
I.
Early vaccination samples
US
Bacterial Richness
Bacterial Faith's PD
Placebo
Vaccine
ns
K.
Early vaccination samples
US
ADONIS
p = 0.315
Axis.1 [39.5%]
Axis.2 [14.9%]
Vaccine Group
Placebo
Vaccine
J.
Late vaccination samples
RW
US
Bacterial Richness
Bacterial Faith's PD
Placebo
Vaccine
ns
L.
Late vaccination samples
RW
US
ADONIS
p = 0.683
p = 0.695
Axis.1 [55.2%]
Axis.2 [15%]
Axis.1 [40.3%]
Axis.2 [19.2%]
Vaccine Group
Placebo
Vaccine

Supplemental Figure 2. Ad26-based HIV-1 vaccination regimens do not significantly alter the bacterial or bacteriophage microbiomes between early and late post-vaccination time-points. Bacteriophage contigs obtained from virome sequencing performed on matched early-late samples from US and Uganda (UG) were analyzed. **(A and B)** Bacteriophage richness and Shannon diversity were pair-matched between early (week 2) and late (week 26) post-vaccination samples from **(A)** US and **(B)** UG. **(C and D)** Dimensional reduction (NMDS) of the Bray-Curtis distances was performed for bacteriophage identified in samples from **(C)** US and **(D)** UG, with samples colored by timepoint. 16S rRNA amplicon sequencing data and bacteriophage contigs from virome sequencing data from placebo and vaccine groups at early (US only) or late (US and Rwanda) timepoints were compared. **(E and F)** Bacterial richness and Faith's phylogenetic diversity (PD) were compared between placebo and vaccine group samples at **(E)** early and **(F)** late post-vaccination timepoints. Principal coordinates analysis (PCoA) using weighted UniFrac distances using 16S rRNA amplicons comparing placebo and vaccine groups at **(G)** early (US) and **(H)** late timepoints (US and Rwanda). Bacteriophage richness and Shannon diversity were compared between placebo and vaccine group samples at **(I)** early (US) and **(J)** late timepoints (US and Rwanda). **(K and L)** Dimensional reduction (NMDS) of the Bray-Curtis distances for bacteriophage contigs in samples from **(K)** early (US) and **(L)** late timepoints (US and Rwanda). Statistical significance for paired samples was determined using the paired Wilcoxon sign-ranked test. Differences between groups in PCoA and NMDS plots was assessed using PERMANOVA. ns = not significant.

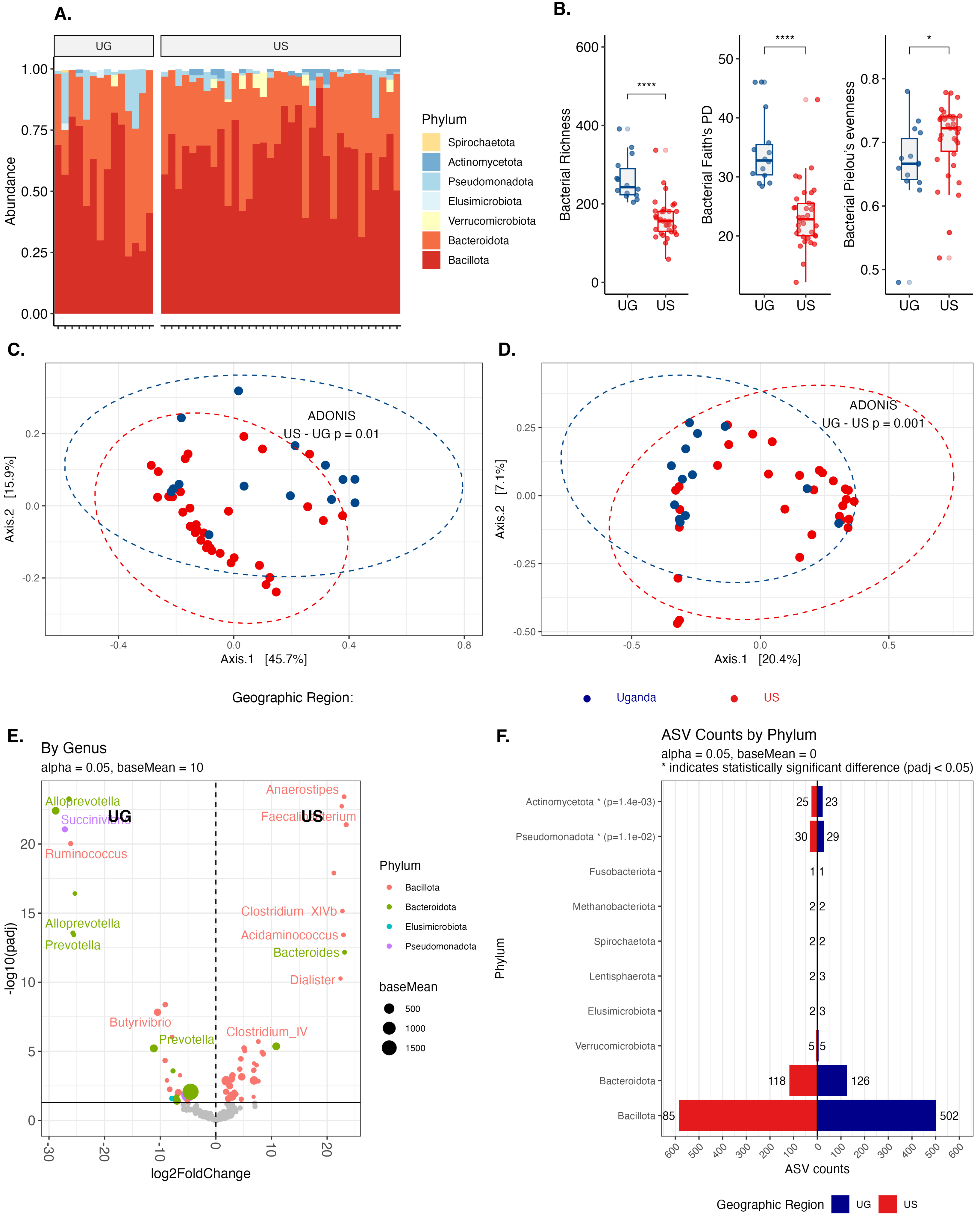

A.
UG
US
Abundance
1.00
0.75
0.50
0.25
0.00
Phylum
Spirochaetota
Actinomycetota
Pseudomonadota
Elusimicrobiota
Verrucomicrobiota
Bacteroidota
Bacillota
B.
Bacterial Richness
Bacterial Faith's PD
Bacterial Pielou's evenness
****
****
*
UG
US
C.
ADONIS
US - UG p = 0.01
Axis.1 [45.7%]
Axis.2 [15.9%]
D.
ADONIS
UG - US p = 0.001
Axis.1 [20.4%]
Axis.2 [7.1%]
Geographic Region:
Uganda
US
E.
By Genus
alpha = 0.05, baseMean = 10
Alloprevotella
Succinivibrio
UG
Ruminococcus
Alloprevotella
Prevotella
Butyrivibrio
Prevotella
Anaerostipes
Faecalibacterium
US
Clostridium_XIVb
Acidaminococcus
Bacteroides
Dialister
Clostridium_IV
-log10(padj)
log2FoldChange
Phylum
Bacillota
Bacteroidota
Elusimicrobiota
Pseudomonadota
baseMean
500
1000
1500
F.
ASV Counts by Phylum
alpha = 0.05, baseMean = 0
* indicates statistically significant difference (padj < 0.05)
Actinomycetota * (p=1.4e-03)
Pseudomonadota * (p=1.1e-02)
Fusobacteriota
Methanobacteriota
Spirochaetota
Lentisphaerota
Elusimicrobiota
Verrucomicrobiota
Bacteroidota
Bacillota
Phylum
ASV counts
Geographic Region
UG
US

Supplemental Figure 3. Geography is a major driver of differences in enteric bacterial communities early post-vaccination in the US and Uganda. 16S rRNA gene amplicon analysis was performed on fecal samples collected from individuals from US and Uganda (UG) at the early (2 week) post-vaccination timepoint. **(A)** Relative abundance of bacterial taxa at the phylum level for samples from individuals from US and UG. Each vertical bar corresponds to a study participant. **(B)** Bacterial richness Faith's phylogenetic diversity and Pielou's Evenness. (PD). **(C)** Principal coordinates analysis (PCoA) using weighted UniFrac distances. **(D)** Dimensional reduction (NMDS) of the Bray-Curtis distances for bacteriophage contigs. **(E and F)** DESeq2 analysis and phylum level summary counts of taxa differentially abundant between UG and US. Statistical significance in alpha diversity between regions was determined using the Dunn's Test and adjusted with the Benjamini-Hochberg procedure. Differences between groups in PCoA and NMDS plots was assessed using PERMANOVA. ****$P < 0.0001$; ***$P < 0.001$; **$P < 0.01$; *$P < 0.05$; ns = not significant.

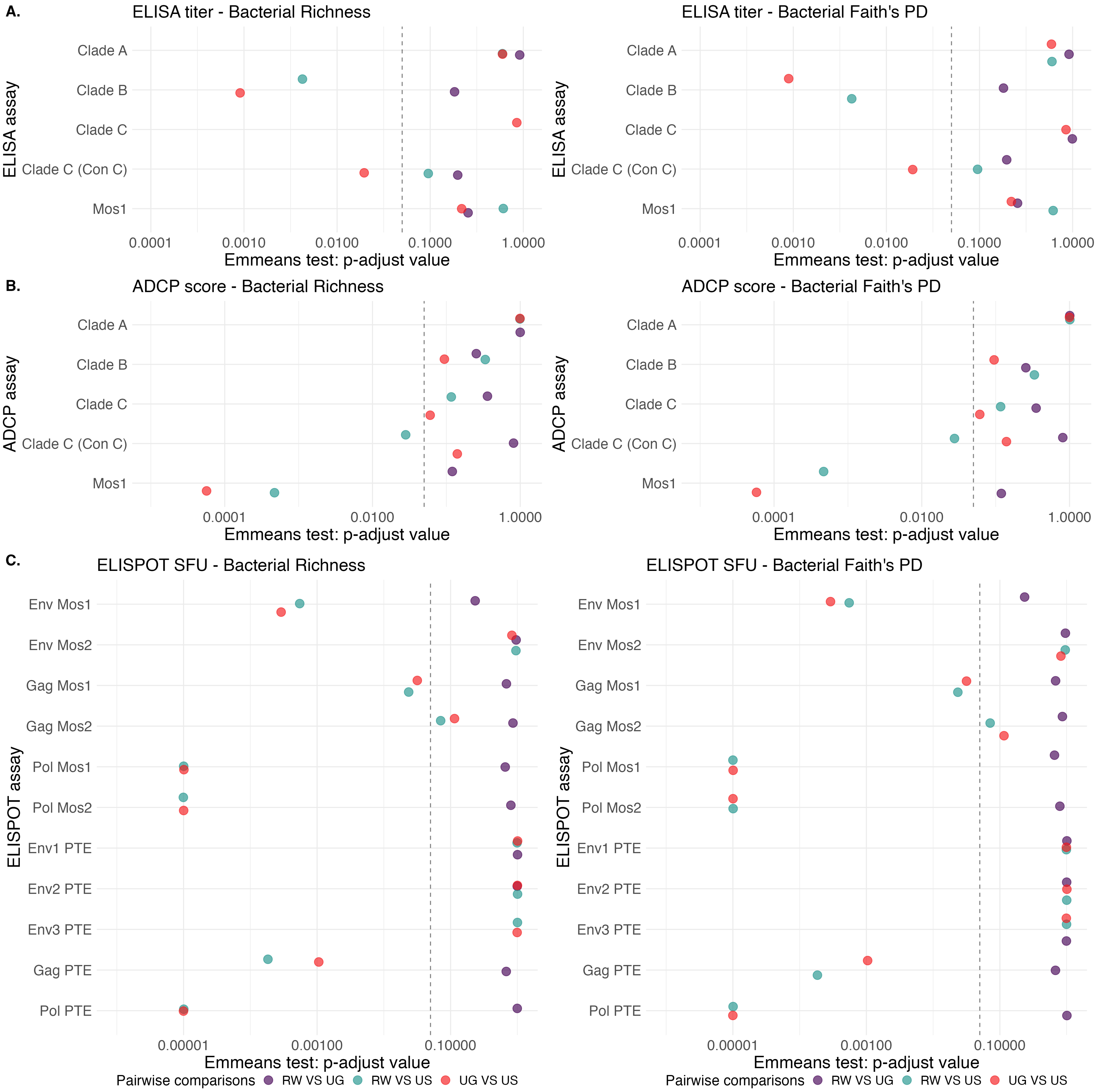
A.
ELISA titer - Bacterial Richness
ELISA titer - Bacterial Faith's PD
ELISA assay
Clade A
Clade B
Clade C
Clade C (Con C)
Mos1
0.0001
0.0010
0.0100
0.1000
1.0000
Emmeans test: p-adjust value
B.
ADCP score - Bacterial Richness
ADCP score - Bacterial Faith's PD
ADCP assay
0.0001
0.0100
1.0000
C.
ELISPOT SFU - Bacterial Richness
ELISPOT SFU - Bacterial Faith's PD
ELISPOT assay
Env Mos1
Env Mos2
Gag Mos1
Gag Mos2
Pol Mos1
Pol Mos2
Env1 PTE
Env2 PTE
Env3 PTE
Gag PTE
Pol PTE
0.00001
0.00100
0.10000
Pairwise comparisons
RW VS UG
RW VS US
UG VS US

Supplemental Figure 4. Summary of significance values (p-values) from multiple comparisons between bacterial alpha diversity and Ad26-based HIV-1 clade and antigen specific immune measures. Complete set of adjusted p-values obtained when performing analysis of covariance (ANCOVA) for both bacterial richness and Faith's phylogenetic diversity (PD) compared to **(A)** ELISA titer, **(B)** ADCP score, or **(C)** ELISpot responses between each region. Vertical dashed line indicates p-value of 0.05. ****$P < 0.0001$; ***$P < 0.001$; **$P < 0.01$; *$P < 0.05$; ns = not significant.
