# Supplementary material for: Associations between the microbiome and immune responses to an adenovirus-based HIV-1 candidate vaccine are distinct between African and US cohorts
Source: mSystems. 2026 Jan 15;11(2):e01435-25. doi: 10.1128/msystems.01435-25 (PMC12911364; doi:10.1128/msystems.01435-25)
Supplement: Supplemental Figures Part 2 — Figures S5 to S7. [file msystems.01435-25-s0002.pdf]

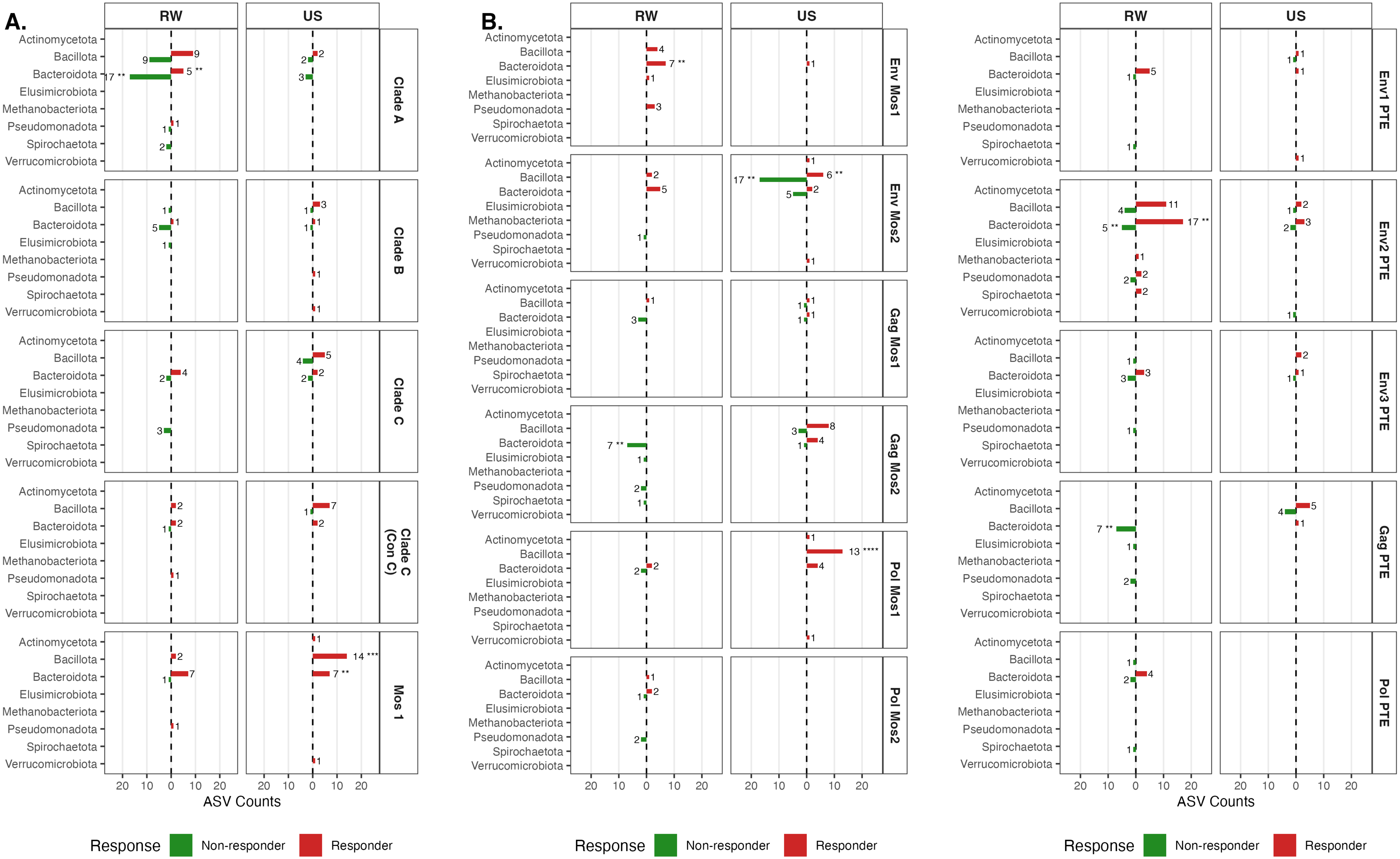

A.
B.
RW
US
Clade A
Clade B
Clade C
Clade C (Con C)
Mos 1
Env Mos1
Env Mos2
Gag Mos1
Gag Mos2
Pol Mos1
Pol Mos2
Env1 PTE
Env2 PTE
Env3 PTE
Gag PTE
Pol PTE
Actinomycetota
Bacillota
Bacteroidota
Elusimicrobiota
Methanobacteriota
Pseudomonadota
Spirochaetota
Verrucomicrobiota
20 10 0 10 20
ASV Counts
Response
Non-responder
Responder

Supplemental Figure 5. Summary of bacterial ASVs associated with HIV clade and antigen specific immune response within Rwanda and US region. ELISpot Summary plot showing the bacterial taxa significantly associated with responder or non-responder samples for **(A)** ADCP and **(B)** ELISpot assays. Vaccine response was defined as value more than threshold (if baseline is <threshold or is missing); otherwise, it was defined as value with a three-time increase from baseline (if baseline is ≥threshold) for Env-specific gp140 antibody-dependent phagocytic cells or antibody titers (ADCP). Vaccine response was defined as geometric mean titers of greater than 200 spot forming cells to one or more HIV antigens (ELISpot) as described previously [21,22,42-44]. Fisher's exact test was used to compare the counts of taxa between different responses group. ****$P < 0.0001$; ***$P < 0.001$; **$P < 0.01$; *$P < 0.05$.****$P < 0.0001$; ***$P < 0.001$; **$P < 0.01$; *$P < 0.05$.

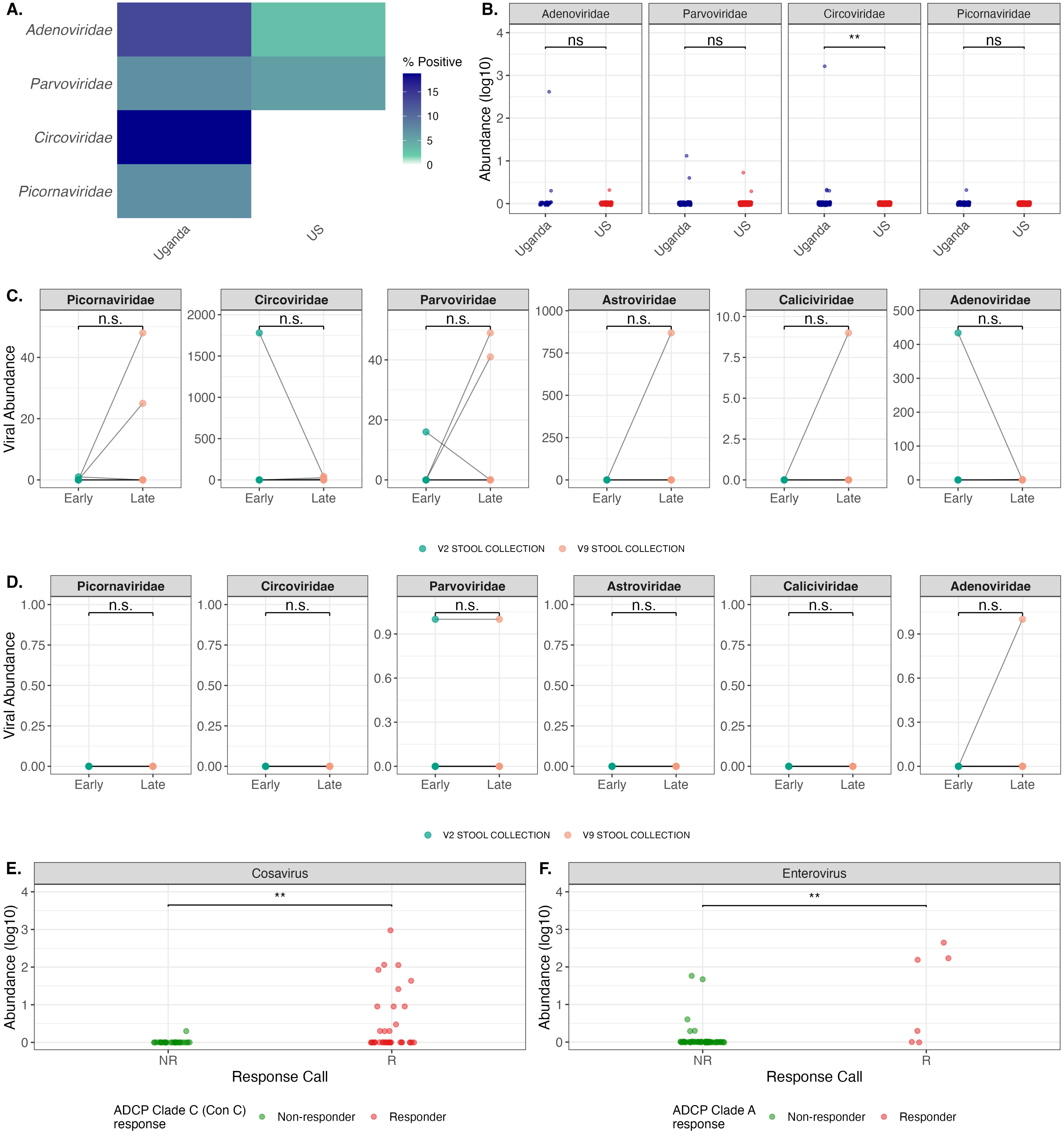

A.
Adenoviridae
Parvoviridae
Circoviridae
Picornaviridae
Uganda
US
% Positive
15
10
5
0
B.
Adenoviridae
Parvoviridae
Circoviridae
Picornaviridae
ns
**
Abundance (log10)
C.
Picornaviridae
Circoviridae
Parvoviridae
Astroviridae
Caliciviridae
Adenoviridae
n.s.
Viral Abundance
Early
Late
V2 STOOL COLLECTION
V9 STOOL COLLECTION
D.
Picornaviridae
Circoviridae
Parvoviridae
Astroviridae
Caliciviridae
Adenoviridae
n.s.
Viral Abundance
Early
Late
V2 STOOL COLLECTION
V9 STOOL COLLECTION
E.
Cosavirus
F.
Enterovirus
**
Abundance (log10)
NR
R
Response Call
ADCP Clade C (Con C) response
Non-responder
Responder
ADCP Clade A response
Non-responder
Responder

Supplemental Figure 6. Ad26-based HIV-1 vaccination does not alter vertebrate viruses. (A) Vertebrate viral families identified from Uganda and US samples at early (week 2) post-vaccination timepoint. **(B)** Comparison of the abundances of each viral family between regions at early (week 2) post-vaccination timepoint. Significance between regions was determined using the Dunn's test, adjusted with Holm. Pair-matched comparisons of viral family abundances between early (week 2) and late (week 26) post-vaccination time-points from **(C)** US and **(D)** Uganda. **(E)** Abundance of *Cosavirus C* in ADCP clade C (Con C) responders and non-responders from Rwanda. **(F)** Abundance of non-polio *Enterovirus C* in ADCP clade A responders and non-responders from Rwanda. Significance between regions and immune responses was determined using the Wilcoxon signed-rank test. Significance between time-points was determined using a paired Wilcoxon signed-rank test. ****$P < 0.0001$; ***$P < 0.001$; **$P < 0.01$; *$P < 0.05$; ns = not significant.

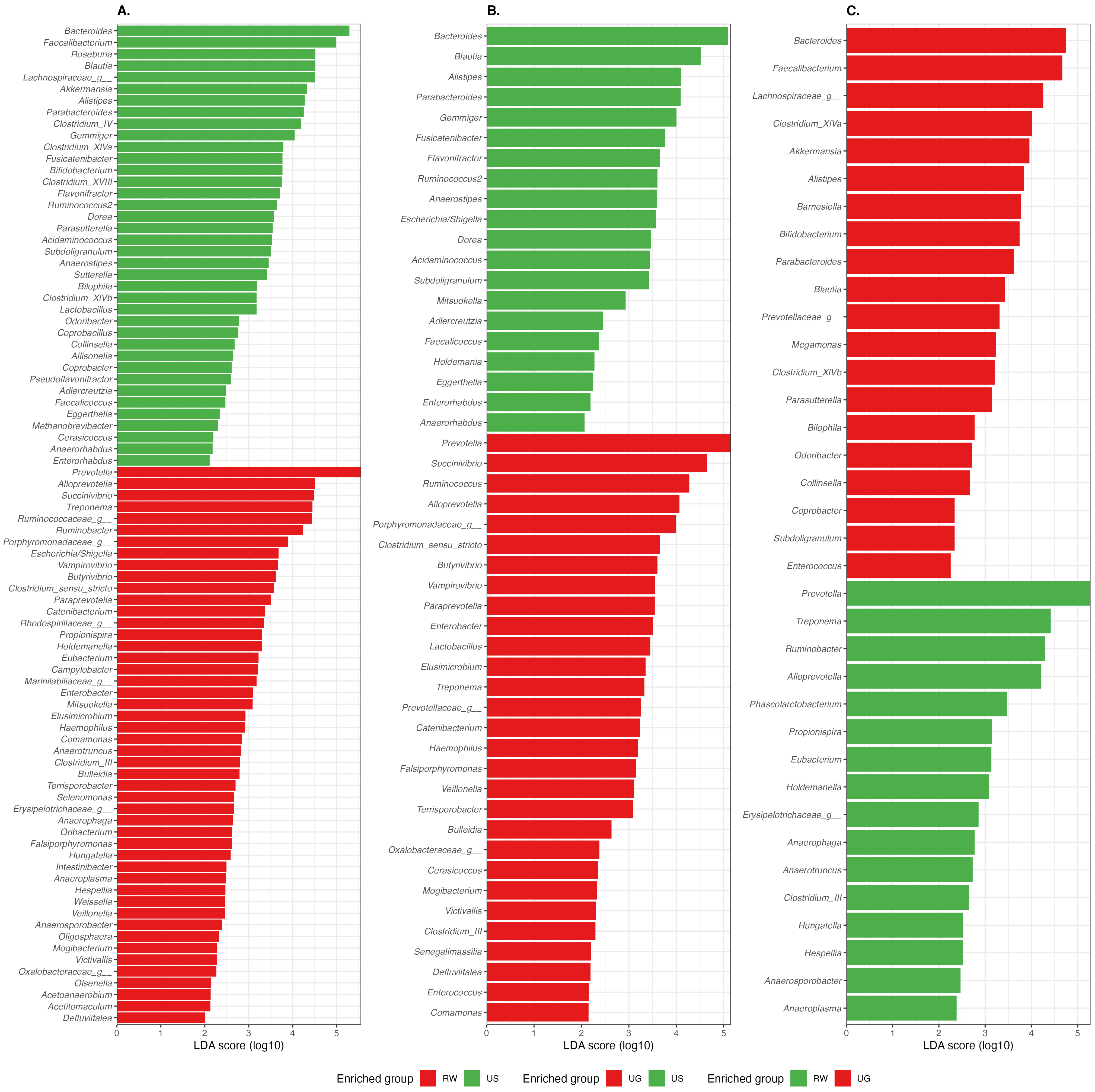

A.
B.
C.
Bacteroides
Faecalibacterium
Roseburia
Blautia
Lachnospiraceae_g__
Akkermansia
Alistipes
Parabacteroides
Clostridium_IV
Gemmiger
Clostridium_XlVa
Fusicatenibacter
Bifidobacterium
Clostridium_XVIII
Flavonifractor
Ruminococcus2
Dorea
Parasutterella
Acidaminococcus
Subdoligranulum
Anaerostipes
Sutterella
Bilophila
Clostridium_XlVb
Lactobacillus
Odoribacter
Coprobacillus
Collinsella
Allisonella
Coprobacter
Pseudoflavonifractor
Adlercreutzia
Faecalicoccus
Eggerthella
Methanobrevibacter
Cerasicoccus
Anaerorhabdus
Enterorhabdus
Prevotella
Alloprevotella
Succinivibrio
Treponema
Ruminococcaceae_g__
Ruminobacter
Porphyromonadaceae_g__
Escherichia/Shigella
Vampirovibrio
Butyrivibrio
Clostridium_sensu_stricto
Paraprevotella
Catenibacterium
Rhodospirillaceae_g__
Propionispira
Holdemanella
Eubacterium
Campylobacter
Marinilabiliaceae_g__
Enterobacter
Mitsuokella
Elusimicrobium
Haemophilus
Comamonas
Anaerotruncus
Clostridium_III
Bulleidia
Terrisporobacter
Selenomonas
Erysipelotrichaceae_g__
Anaerophaga
Oribacterium
Falsiporphyromonas
Hungatella
Intestinibacter
Anaeroplasma
Hespellia
Weissella
Veillonella
Anaerosporobacter
Oligosphaera
Mogibacterium
Victivallis
Oxalobacteraceae_g__
Olsenella
Acetoanaerobium
Acetitomaculum
Defluviitalea
0 1 2 3 4 5
LDA score (log10)
Bacteroides
Blautia
Alistipes
Parabacteroides
Gemmiger
Fusicatenibacter
Flavonifractor
Ruminococcus2
Anaerostipes
Escherichia/Shigella
Dorea
Acidaminococcus
Subdoligranulum
Mitsuokella
Adlercreutzia
Faecalicoccus
Holdemania
Eggerthella
Enterorhabdus
Anaerorhabdus
Prevotella
Succinivibrio
Ruminococcus
Alloprevotella
Porphyromonadaceae_g__
Clostridium_sensu_stricto
Butyrivibrio
Vampirovibrio
Paraprevotella
Enterobacter
Lactobacillus
Elusimicrobium
Treponema
Prevotellaceae_g__
Catenibacterium
Haemophilus
Falsiporphyromonas
Veillonella
Terrisporobacter
Bulleidia
Oxalobacteraceae_g__
Cerasicoccus
Mogibacterium
Victivallis
Clostridium_III
Senegalimassilia
Defluviitalea
Enterococcus
Comamonas
0 1 2 3 4 5
LDA score (log10)
Bacteroides
Faecalibacterium
Lachnospiraceae_g__
Clostridium_XlVa
Akkermansia
Alistipes
Barnesiella
Bifidobacterium
Parabacteroides
Blautia
Prevotellaceae_g__
Megamonas
Clostridium_XlVb
Parasutterella
Bilophila
Odoribacter
Collinsella
Coprobacter
Subdoligranulum
Enterococcus
Prevotella
Treponema
Ruminobacter
Alloprevotella
Phascolarctobacterium
Propionispira
Eubacterium
Holdemanella
Erysipelotrichaceae_g__
Anaerophaga
Anaerotruncus
Clostridium_III
Hungatella
Hespellia
Anaerosporobacter
Anaeroplasma
0 1 2 3 4 5
LDA score (log10)
Enriched group RW US
Enriched group UG US
Enriched group RW UG

Suplemental Figure 7: Linear discriminant analysis Effect Size (LEfSe) analysis of bacterial genera across geographic regions.

This figure illustrates differentially abundant bacterial genera identified by LEfSe analysis comparing gut microbiome compositions across three geographic regions (RW, UG, and US), week 26 (late) after vaccination with multivalent adenovirus serotype 26 (Ad26) vectored mosaic HIV-1 vaccines. The horizontal bars represent the LDA scores (log10 transformed) for each bacterial genus, with longer bars indicating stronger statistical significance and biological relevance. **(A)** Comparison between RW (red) and US (green) populations showing distinct bacterial signatures. **(B)** Comparison between UG (red) and US (green) populations highlighting region-specific taxa. **(C)** Comparison between RW (green) and UG (red) populations demonstrating microbiome differences between the two African regions. Genera are ordered by their relative abundance, with taxa significantly enriched in each geographic region colored accordingly.
